# Age-dependent regulation of dendritic spine density and protein expression in Mir324 KO mice

**DOI:** 10.21203/rs.3.rs-3221779/v1

**Published:** 2023-08-07

**Authors:** Emma V Parkins, John M Burwinkel, Ruvi Ranatunga, Sarah Yaser, Yueh-Chiang Hu, Durgesh Tiwari, Christina Gross

**Affiliations:** Cincinnati Children’s Hospital Medical Center

**Keywords:** microRNA, dendritic spines, neuronal development, miR-324-5p, Kv4.2

## Abstract

Dendritic spines are small, dynamic protrusions along the dendrite that comprise more than 90% of excitatory connections in the brain, making them essential sites for neuronal communication. These synaptic sites change throughout the process of development, reducing in density and shifting morphology as synapses are refined. One important class of dendritic spine regulators is microRNA (miRNA), small noncoding RNAs that post-transcriptionally regulate gene expression. Several studies suggest that miRNA-324-5p regulates dendritic spine formation. In addition, we have previously shown that miR-324-5p plays a role in seizure and long-term potentiation, both of which involve dendritic spine changes. In this study, we aimed to characterize the role of miRNA-324-5p in developmental spine regulation by assessing the effect of *Mir324* knockout (KO) on dendritic spine density and expression of a subset of dendritic proteins at select developmental time points. We show that miR-324-5p expression is developmentally regulated and peaks at four weeks of age. We demonstrate that loss of miR-324-5p expression leads to differential changes in both target protein expression and spine density at different time points during development, disrupting the pattern of spine density changes and leading to a premature loss of dendritic spines in KO mice, which is compensated later. Our findings indicate that miR-324-5p plays a role in synaptic refinement across development. Additionally, our data illustrate the importance of context in the study of miRNA, as regulation by and/or of miRNA can vary dramatically across development and in disease.

## Introduction

Synapses are overproduced and pruned across development in a tightly orchestrated process involving multiple regulators (Bredy et al., 2011; Dogini et al., 2013; Edbauer et al., 2010; Riccomagno & Kolodkin, 2015; Sorra & Harris, 2000; Thomas et al., 2018) whose mechanisms vary as time point-specific changes in miRNA expression occur (Moreau et al., 2013). Most excitatory synapses in the brain are formed on small protrusions along the dendrite called dendritic spines. Dendritic spines are highly plastic structures with complex, localized regulatory networks (Biever et al., 2019), and they characteristically change across development, reducing in density and shifting morphology as synapses are refined (Riccomagno & Kolodkin, 2015; Sorra & Harris, 2000).

Understanding what regulates dendritic spine changes and characterizing mechanisms of spine regulation are essential steps toward managing their dysregulation in disease. One important class of dendritic spine regulators is microRNA. MiRNAs are small noncoding RNAs that post-transcriptionally regulate gene expression. Subcellular expression studies of microRNA have identified several that are expressed in and around dendritic spines (Corbin et al., 2009; Hu & Li, 2017; Koester & Dougherty, 2022; Smalheiser & Lugli, 2009), indicating a fine-tuned environment of miRNA regulation at these sites. One such microRNA is miR-324-5p. MiR-324-5p is primarily expressed in brain tissue (Ludwig et al., 2016) and targets several dendritic spine-localized proteins (Gross et al., 2016; Tiwari et al., 2019). Studies suggest that it plays an essential role modulating neuronal excitability (Hayman et al., 2021), seizures (Gross et al., 2016; Tiwari et al., 2019), and long-term potentiation (LTP) (Parkins et al., 2023).

In this study, we aim to characterize the role of miR-324-5p in developmental spine regulation by assessing the effect of *Mir324* knockout (KO) on dendritic spine density and protein expression at select developmental time points. Some of the earliest studies of microRNAs identified important roles in neurodevelopment (Giusti et al., 2014; Hu & Li, 2017; Schratt et al., 2006) as well as in the adult brain (Bredy et al., 2011; Brennan & Henshall, 2020; Smalheiser & Lugli, 2009). MicroRNA may operate very differently within the synapse from early development to adulthood. The synaptic environment changes drastically throughout development, altering the context in which regulators like microRNAs operate, the availability of mRNA targets, and thus likely changing their function. In keeping with this, we found that the effect of *Mir324* KO on both dendritic spine density and target protein and mRNA expression varied across developmental time points. MiR-324-5p is differentially expressed across development, increasing after birth before peaking in expression around postnatal day (PD) 28. The effect of *Mir324* KO on dendritic spine density was greatest following this peak in expression, resulting in an early reduction in spine density relative to wildtype (WT). Overall, this study demonstrates that microRNA-mediated regulation of dendritic spines varies by age. In a broader sense, our results show that it is essential that researchers consider development and age in the study of microRNA function, as we demonstrate that microRNA function can vary significantly between relatively close time points.

## Materials and Methods

### Animals

All animal procedures were approved by the Institutional Animal Care and Use Committee of CCHMC and complied with the Guideline for the Care and Use of Laboratory Animals, under the Animal Welfare Assurance number D16-00068.

*Mir324* KO mice were generated by the CCHMC Transgenic Animal and Genome Editing Core Facility using CRISPR/Cas9 gene editing of C57BL/6N mice as previously described (Parkins et al., 2023). Mice in this study were obtained from either *Mir324* knockout (KO) pairs or *Mir324*-strain wild-type (WT)/C57BL/6J WT mice. *Mir324* KO and WT breeding mice were selected from litters from *Mir324* heterozygous mice mated with C57BL/6J mice (RRID:IMSR_JAX:000664). Pups were weaned at P28 and were housed with same sex littermates (minimum 2 and maximum 4 per cage) in a standard cage with food and water provided ad libitum. All cages were enriched with a standard mouse igloo. Mice were maintained on a standard 14:10 hour light:dark cycle and all experiments were performed during the light cycle.

### Golgi stain

Dendritic spines of CA1 pyramidal neurons (bregma level – 1.9 to −2.2) were assessed using Rapid GolgiStain Kit (FD Neurotechnologies, MD). Harvested brains were subjected to Golgi impregnation, sectioned at 120 μm thickness, and stained as per manufacturer’s protocol. Sections were imaged using a Nikon inverted confocal microscope with 4X, 20X, and 60X/NA1.4 oil immersion lenses. Dendritic spines of CA1 pyramidal neurons located across the CA1 were assessed on secondary dendrites located at least 50 μm from the cell body. For earlier time points (PD 14–28), dendrite segments 20–80 μm in length were included in analysis; for all following time points, dendrites were between 60–120 μm. On average, 4–5 dendrites from 3 neurons were assessed per mouse by experimenters blinded to genotype and sex. Spines were manually counted on ImageJ (NIH, RRID:SCR_003070) and spine counts of all neurons from each WT and *Mir324* KO mice of the same sex from each litter were pooled and counted as n = 1 for statistical analysis to avoid litter effects. Thus, each data point represents the average of sex- and genotype-matched mice from one litter.

### Nissl staining and hippocampal measurements

Following deep anesthesia with at least 200 mg/kg pentobarbital, mice > 28 days old were intracardially perfused with 2% paraformaldehyde (PFA). Because several mice in this age group were found to be too small to effectively perfuse (weighing < 8 g), mice aged PD 14 and 28 were anesthetized with CO_2_ following CCHMC IACUC guidelines. Whole brains were removed and preserved in 4% PFA overnight at 4 C prior to cryoprotection with sucrose solution (24 hr 10%, 20%, and 30% sucrose at 4 C) and cryopreservation at −80 C. Brains were embedded in Tissue-Tek OCT compound and sectioned at 20 μm, then stained with Neurotrace (435/455, Fisher Scientific, N21479) according to the product’s protocol. Slides were imaged at 10X and measurements of the hippocampus were obtained via Nikon Elements (Tokyo, Japan, RRID:SCR_014329) and ImageJ (RRID:SCR_003070) software and assessed individually. Five hippocampal measurements (H1-5) were completed as in (White et al., 2020). Briefly, measurement H4 transects the dentate gyrus from dentate granule cell dendrites at the apex to the end of the blade. H5 bisects H4 and extends across the shells of the dentate, from dendrites at the supramedial to infrapyramidal blades. H1-3 evenly transect H4, originating beneath the granule cell layer and ending at the tip of basal dendrites of CA1 pyramidal cells. Brain sections were assessed for each mouse at the approximate bregma level of −1.7 mm. Each data point represents the average of sex-matched mice from one litter.

### SDS-PAGE and Western blot analysis

Whole hippocampi were used to assess protein expression. Protein concentration was determined using Bio-Rad Protein Assay Dye (Hercules, California, USA; Cat: 5000006). Samples were mixed with SDS sample buffer and 10 μ of protein was loaded in duplicate on SDS-PAGE gels, then transferred to PVDF Transfer Membrane (Millipore Sigma, Darmstadt, Germany). Membranes were blocked using 5% milk for 1–2 hours. Antibodies were diluted in 1% Tween in PBS or 5% milk prepared in 1% Tween in PBS and incubated overnight at 4°C. Membranes were then washed and incubated with secondary antibody, either Rabbit IgG HRP Linked Whole Antibody (Millipore Sigma, Darmstadt, Germany; Cat: GENA934) or Mouse IgG HRP Linked Whole Antibody (Millipore Sigma, Darmstadt, Germany; Cat: NXA931V). Signals were detected with enhanced chemiluminescence using Pierce ECL Blotting Substrate (Thermo Scientific, Carlsbad, CA, USA, Cat:32106). If a second detection was needed, blots were stripped using Restore Western Blot Stripping Buffer (Thermo Scientific, Carlsbad, CA, USA, Cat:21059), blocked again in 5% milk, and incubated overnight with the desired antibody.

Signal intensities of proteins were normalized to GAPDH signal on the same blot. Duplicates were averaged for each data point. Protein-specific signals on Western blots were quantified densitometrically using NIH ImageJ software (Bethesda, Maryland, USA).

### RNA isolation and qRT-PCR

RNA was extracted using Trizol^®^ (Life Technologies, Carlsbad, CA). Quality and quantity of mRNA was measured using a Nanodrop Spectrophotometer (Thermo Fisher Scientific, Waltham, MA) or BioTek Cytation Imaging Microplate Reader (BioTek, Winooski, VT) and RNA dilutions were made in nuclease-free water.

cDNA was generated using 1μ RNA with the High Capacity RNA-to-cDNA Kit (Applied Biosystems, Foster City, CA) for mRNA, or qScript^™^ microRNA cDNA synthesis kit (Quanta BioSciences, Gaithersburg, MD) for miRNA, followed by SYBR green quantitative real-time PCR (iTaq Universal SYBR green supermix, Bio-Rad Laboratories, Hercules, CA). Individual qPCRs were carried out on the QuantStudio 3 Real-Time PCR System (Applied Biosystems, Foster City, CA Relative changes were quantified using the comparative cycle threshold method (2 – ΔCT). For mRNA, expression was normalized to GAPDH. For miRNA, expression was normalized to miR-191.

### Immunohistochemistry, imaging, and colocalization analysis

Following deep anesthesia with at least 200 mg/kg pentobarbital, mice were intracardially perfused with 2% paraformaldehyde (PFA). Whole brains were removed and preserved in 4% PFA overnight at 4 C before undergoing cryoprotection with sucrose solution (24 hr each in 10%, 20%, and 30% sucrose at 4 C) and cryopreservation at −80 C. Brains were embedded in Tissue-Tek OCT compound and sectioned at 20 μm. Tissue was permeabilized and blocked against nonspecific antibody binding over 2–4 hrs in 0.5% Triton X (Rohm and Haas Company), 3% Fetal Bovine Serum (Fisher Scientific, Cat #10082147), and 3% Normal Donkey Serum (Fisher Scientific, Cat #5664605ML) in 1x PBS (Fisher Scientific, Cat #50550429). Primary and secondary antibody were applied for 24 hours each at 4 C. Tissue was mounted in ProLong Diamond Antifade Mountant (Life Technology, Cat #P36961).

Sections (500 x 500 μm) within the apical dendrites of the hippocampal CA1 were imaged using a Nikon A1R LUNV confocal microscope with the 60X water immersion lens. Shot noise was removed from images using the Nikon NIS Denoise.ai (Nikon Instruments Inc.). Images were cropped into 3-frame z-stacks (for total depth of 0.6 um). Colocalization analysis was performed on each frame in NIS Elements (RRID:SCR_014329) and reported as Manders’ coefficient (Manders et al., 1993), the proportion of PSD95 staining that overlapped with vGlut1. Additionally, the proportion of colocalized PSD95, reported as Manders’ coefficient K1, and the proportion of colocalized vGlut1, reported as Manders’ coefficient K2, were assessed in NIS Elements. Finally, to account for any differences in background staining, Pearson’s correlation coefficient was analyzed.

### Antibodies and primers:

The following antibodies were used:

#### Western blot:

Kv4.2 rabbit polyclonal anti-Kv4.2 (Proteintech Group, Rosemont, IL Cat# 21298-1-AP, RRID:AB_10733102), MAP2 (high molecular weight) rabbit polyclonal (Millipore Cat# AB5622, RRID:AB_91939), GAPDH mouse monoclonal (Abcam Inc Cat# AB9484, RRID:AB_307274), Anti-PSD-95 MAGUK scaffold protein mouse monoclonal (Antibodies Incorporated Cat# 75–348, RRID:AB_2315909), Rabbit IgG HRP Linked Whole Antibody (Millipore Sigma, Darmstadt, Germany; Cat# GENA934), Mouse IgG HRP Linked Whole Antibody (Millipore Sigma, Darmstadt, Germany; Cat# NXA931V).

#### IHC:

PSD95 rabbit polyclonal (Synaptic Systems, Cat# 124003, RRID:AB_2725761), VGlut1 guinea pig polyclonal (Synaptic Systems Cat# 135 304, RRID:AB_887878), goat anti-guinea pig IgG (H + L) Alexa Fluor^™^ 647 (Thermo Fisher Scientific Cat# A-21450, RRID:AB_141882), goat anti-rabbit IgG (H + L) Alexa Fluor^™^ 594 (Thermo Fisher Scientific Cat# A-11012, RRID:AB_2534079).

The following qRT-PCR primers were used:

GAPDH for: GGGTTCCTATAAATACGGACTGC; GAPDH rev: CCATTTTGTCTACGGGACGA; Kv4.2 for: GCTTTGAGACACAGCACCAC; Kv4.2 rev: TGTTCATCGACAAACTCATGG; PSD95 for: 5TCTGTGCGAGAGGTAGCAGA; PSD95 rev: AAGCACTCCGTGAACTCCTG; MAP2 for: CTGGACATCAGCCTCACTCA; MAP2 rev: AATAGGTGCCCTGTGACCTG; miR-324-5p: CGCATCCCCTAGGGCATTGGTGT.

### Statistics:

All analyses were performed by experimenters blinded to genotype, age, and sex. Appropriate parametric or nonparametric statistical tests (indicated in figure legends) were determined and run using GraphPad Prism version 8 (GraphPad Software, San Francisco, CA). Normality was tested using the Shapiro-Wilk test, and data with unequal variance was assessed using nonparametric methods. Outliers were identified as ± 2*SD from mean and removed. Sample sizes were determined using R (R Core Team 2020) and published or preliminary effect sizes. Significance level was set to α < 0.05. Each data point represents the pooled data of sex-matched mice from each litter. Data for postnatal day 45–60 mice includes data from PD 60 previously reported in (Parkins et al., 2023).

Our data showed no sex effects in *Mir324* KO mice in spine density (Supplemental Table 1), protein expression (Supplemental Table 2), or mRNA expression (Supplemental Table 3). Both male and female mice were used in each experiment.

## Results

### MiR-324-5p is differentially expressed at select developmental time points

**Figure 1:**
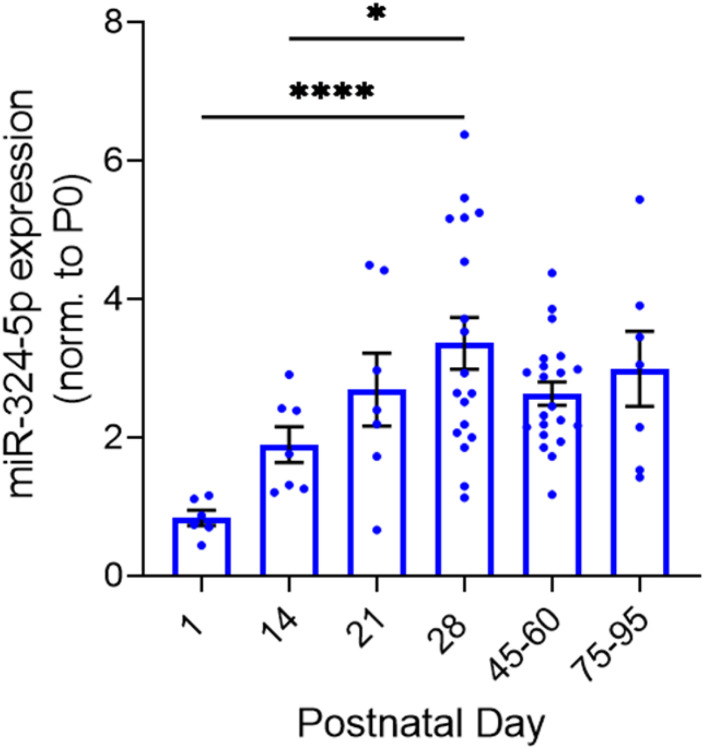
Hippocampal miR-324-5p expression across development. Whole hippocampi were dissected at time points across development (indicated on x-axis). Hippocampal miR-324-5p expression increases after postnatal day 0, peaking at postnatal day 28 (Welch’s one-way ANOVA, **p<0.001; Dunnett’s T3 multiple comparisons, *p=0.0188, ****p<0.0001; n (PD 1)=6, n (PD 14)=7, n (PD 21)=7, n (PD 28)=14, n (PD 45-60)=21, n (PD 75-95)=7). Error bars are SEM.

Measuring microRNA expression in whole hippocampal lysate of wildtype mice at various developmental timepoints, we found that miR-324-5p expression varies significantly across development (Fig. 1; Welch’s one-way ANOVA, p < 0.0001; avg n = 11 mice per time point). Following birth, miR-324-5p expression increases until reaching a peak in expression around postnatal day 28 (PD 28). After PD 28, miR-324-5p expression begins to plateau (Dunnett’s T3, ****p(28 vs 1) < 0.0001, *p(28 vs 14) = 0.019, p(28 vs 21) = 0.83, p(28 vs 45–60) = 0.365, p(28 vs 75+) = 0.984). MiR-324-5p is differentially expressed in the hippocampus across development, indicating that this miRNA functions in normal hippocampal development.

### Male Mir324 KO mice have reduced body weight in adolescence

**Figure 2:**
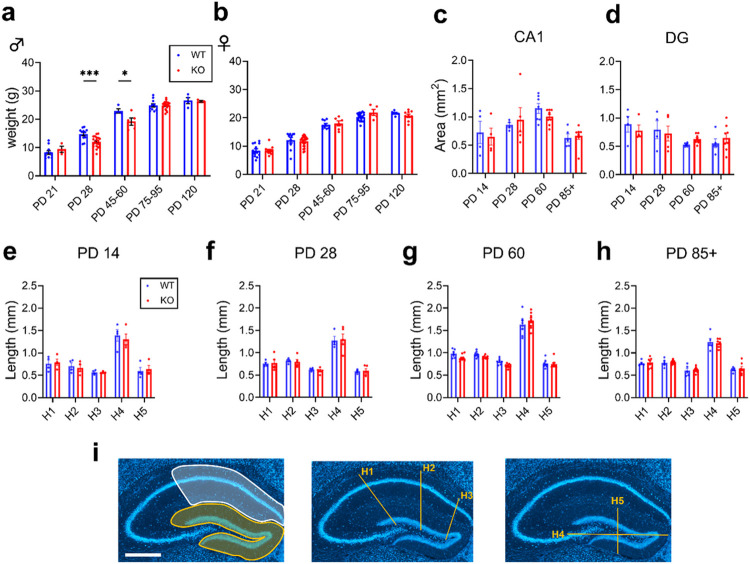
Male *Mir324* KO mice have reduced body weight in adolescence; hippocampal morphology varies by age but not genotype. (A) Weight of *Mir324* WT (blue) and KO (red) male mice across development. *Mir324* KO males have reduced body weight at PD 28 and PD 45-60 (two-way ANOVA, ****p(age)<0.0001, *p(genotype)=0.0269, **p(interaction)=0.006; Sidak’s multiple comparisons, ***p(PD 28)=0.0008, *p(PD 45-60)=0.0215; n(PD 21) WT=10, KO=3, n(PD 28): WT=11, KO=20, n(PD 60): WT=4, KO=5, n(PD 75-95+): WT=10, KO=20 , n(PD 120) WT=4, KO=3). (B) In female mice, weight is affected by age but not genotype (two-way ANOVA, ****p(age)<0.0001, p(genotype)=0.704, p(interaction)=0.418 n(PD 21) WT=13, KO=12, n(PD 28): WT=10, KO=25, n(PD 60): WT=9, KO=6, n(PD 75-95+): WT=18, KO=5 , n(PD 120) WT=6, KO=9). (C) Area of the CA1 (C) and dentate gyrus (D) vary significantly by age but not genotype (C: two-way ANOVA, ***p(age)=0.0003, p(genotype)=0.768, p(interaction)=0.6594; D: two-way ANOVA, *p(age)=0.0188, p(genotype)=0.9532, p(interaction)=0.5477). (E-H) There is no effect of genotype on hippocampal measurements at PD 14 (E; RM two-way ANOVA, ****p(measurement)<0.0001, p(genotype)=0.8453, p(interaction)=0.9116), PD 28 (F; 2-Way RM ANOVA, ****p(measurement)<0.0001, p(genotype)=0.988, p(interaction)=0.9873), PD 60 (G; 2-Way RM ANOVA, ****p(measurement)<0.0001, p(genotype)=0.1867, p(interaction)=0.086), or PD85 + (H; 2-Way RM ANOVA, ****p(measurement) <0.0001, p(genotype)=0.8281, p(interaction)=0.941; n(PD 14): WT=4, KO=4, n(PD 28): WT=4, KO=5, n(PD 60): WT=6, KO=9, n(PD 85+): WT=5, KO=8). (I) Representative images of hippocampal measurements. In the left panel, the CA1 is highlighted in white and DG in yellow. The other two panels show hippocampal measurements H1-5. Scale bar is 500 μm. Error bars are SEM.

*Mir324* KO does not lead to significant changes in body weight of adult male or female mice (Parkins et al., 2023). To more thoroughly characterize the effect of *Mir324* loss on body weight, we weighed WT and KO mice across development (Fig. 2A,B). Body weight varied significantly by age for both male (2A, two-way ANOVA, ****p(age) < 0.0001) and female (2B, ****p(age) < 0.0001) mice as expected, but genotype only affected male mice (*p(genotype) = 0.0269, **p(interaction) = 0.006). This effect was mainly driven by the period of adolescence, when male *Mir324* KO mice were significantly lower in weight than WT mice (***p(PD28) = 0.0008, *p(PD45-60) = 0.0215).

### Mir324 KO does not affect hippocampal morphology

We next analyzed gross hippocampal morphology across development. We identified no significant changes in any hippocampal morphology measures by genotype (Fig. 2C-I). The area of the CA1 subregion of the hippocampus varied by age but not genotype (Fig. 2C; two-way ANOVA, ***p(age) = 0.0003, p(genotype) = 0.768, p(interaction) = 0.659), with no significant differences by genotype at any age. The same was found for the area of the dentate gyrus (DG) (Fig. 2D; two-way ANOVA, *p(age) = 0.0188, p(genotype) = 0.953, p(interaction) = 0.548). No effect of genotype was found for any of the other hippocampal measures (H1-5) at any time point (Fig. 2F-I; RM two-way ANOVA, all > 0.05). This suggests that miR-324-5p loss does not affect the gross morphology of the hippocampus.

### Mir324 KO leads to a premature reduction in hippocampal dendritic spine density during adolescence

**Figure 3:**
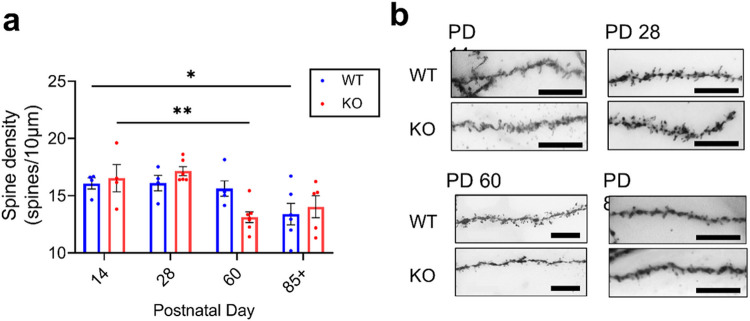
Hippocampal dendritic spine density changes across development depending on *Mir324* genotype. (A) Dendritic spine density varies across development (two-way ANOVA, p(interaction)=0.069, p(age)<0.001, p(genotype)=0.874; n=4-6 mice per group with 3-6 dendrites assessed per mouse, dots represent mice). Pairwise comparisons show that in *Mir324* KO hippocampal neurons, dendritic spine density is significantly reduced relative to PD 14 by PD60 (Dunnett’s multiple comparisons test, p(14 vs 28)=0.884, **p(14 vs 60)=0.0073, p(14 vs 85+)=0.081), whereas in WT this reduction is not significant until PD85+ (Dunnett’s multiple comparisons test, p(14 vs 28)>0.99, p(14 vs 60)=0.958, *p(14 vs 85+)=0.0477). (B) Example images of Golgi-stained dendrites at each time point. Error bars are SEM. Scale bars are 10 μm. No sex differences were found (Supplemental Table 1). N values are listed in Supplemental Table 1.

Dendritic spine density varied significantly by age but not genotype (Fig. 3; two-way ANOVA, ***p(age) = 0.0008, p(genotype) = 0.874, p(interaction) = 0.069). Analysis revealed, however, that the pattern of dendritic spine density change across development varied by genotype, with WT mice showing a reduction in dendritic spine density by PD 85+ (Dunnett’s multiple comparisons test, p(14 vs 28) > 0.99, p(14 vs 60) = 0.958, *p(14 vs 85+) = 0.0477) and KO mice showing an earlier reduction in spine density by PD 60 (Dunnett’s multiple comparisons test, p(14 vs 28) = 0.884, **p(14 vs 60) = 0.0073, p(14 vs 85+) = 0.081). The shift in dendritic spine reduction coincided with the observed peak in miR-324-5p expression at PD 28 (Fig. 1), indicating that miR-324-5p plays an important role in normal dendritic spine refinement.

### Mir324 KO alters hippocampal PSD95 mRNA and protein expression differentially across development

**Figure 4:**
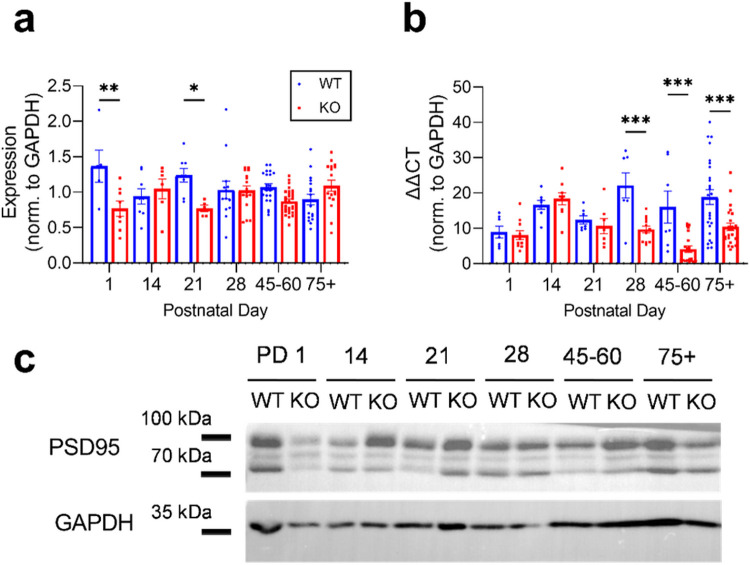
PSD95 protein and mRNA are differentially expressed across development in *Mir324* KO hippocampus. (A) Significant effect of genotype on PSD95 protein expression (two-way ANOVA, p(interaction)=0.0002, p(age)=0.92, p(genotype)=0.0038; Sidak’s multiple comparisons, **p(PD1)=0.0027, *p(PD21)=0.032). (B) Significant interaction between age and genotype on PSD95 mRNA expression (two-way ANOVA, p(interaction) = 0.003, p(age)<0.001, p(genotype)<0.0001; Sidak’s multiple comparisons, ***p(PD 28)=0.001, ***p(PD 45-60)=0.0006, ***p(PD 75+)=0.0001). (C) Western blot example images for WT and KO hippocampal tissue at each timepoint. GAPDH loading control bands are shown at the bottom. Error bars are SEM. Full blots are shown in Supplemental Figure 1, n values and lack of sex differences are shown in Supplemental Tables 2 and 3.

We next used postsynaptic density protein-95 (PSD95), a synaptic scaffolding protein localized to excitatory synapses (Won et al., 2017), to assess potential changes in excitatory synapse composition across development in *Mir324* KO mice. We quantified PSD95 protein (Fig. 4A) and mRNA (Fig. 4B) in hippocampal lysates at select developmental time points. *Mir324* KO led to a significant reduction in PSD95 protein expression overall (two-way ANOVA, p(age) = 0.922, **p(genotype) = 0.0038, ***p(interaction) = 0.0002), with the greatest reductions in the *Mir324* KO compared with WT at PD 1 (Sidak’s multiple comparisons, **p = 0.0027) and PD 21 (*p = 0.032). PSD95 mRNA was also significantly reduced in KO hippocampi (two-way ANOVA, ***p(age) = 0.0003, ****p(genotype) < 0.0001, **p(interaction) = 0.0034), but this effect was driven by reductions later in development, with significantly lower PSD95 mRNA measured at PD 28 (Sidak’s multiple comparisons, **p = 0.001), PD 45–60 (***p = 0.0006), and PD 85+ (***p = 0.0001). Thus, loss of miR-324-5p dysregulates PSD95 protein and mRNA expression differentially across development.

### Kv4.2 protein and mRNA expression in the hippocampus are affected by Mir324 KO in early development

**Figure 5:**
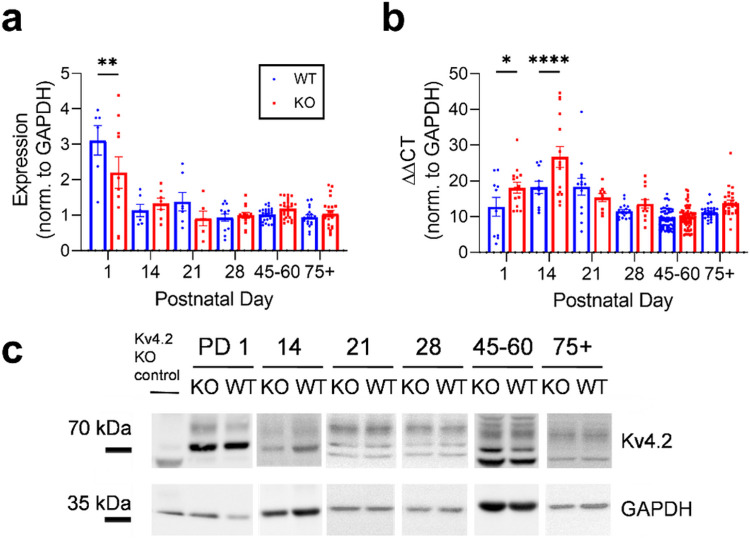
Kv4.2 protein and mRNA are differentially expressed across development in *Mir324* KO hippocampus. (A) Significant interaction between age and genotype on Kv4.2 protein expression (two-way ANOVA, **p(interaction)=0.0061, ****p(age)<0.0001, p(genotype)= 0.112; Sidak’s multiple comparisons, **p(PD1)=0.0042). (B) Interaction between age and genotype on Kv4.2 mRNA expression (two-way ANOVA, ***p(interaction)=0.0003, ****p(age)<0.0001, ****p(genotype)=0.0001; Sidak’s multiple comparisons, *p(PD1)=0.0347, ****p(PD14)<0.0001). (C) Western blot example images for WT and KO hippocampal tissue at each timepoint. Note that different Kv4.2 band patterns for different age points are most likely due to differential phosphorylation of Kv4.2 across development. All bands that were absent in the Kv4.2 KO lane were used for quantification. Error bars are SEM. Full blots are shown in Supplemental Figure 1, n values and lack of sex differences are shown in Supplemental Tables 2 and 3.

Kv4.2 is an A-type potassium channel targeted by miR-324-5p (Gross et al., 2016). Kv4.2 protein expression varies significantly by age, and genotype affects age-dependent changes (Fig. 5A; two-way ANOVA, ***p(age) < 0.0001, p(genotype) = 0.1222, **p(interaction) = 0.006). Regardless of genotype, Kv4.2 protein expression decreased after PD1. At PD1, but not at any other time point tested, KO hippocampi have significantly reduced Kv4.2 protein expression compared with WT hippocampi (Sidak’s multiple comparisons, **p = 0.0042). Kv4.2 mRNA expression varied by both age and genotype (Fig. 5B; 2-way ANOVA, ****p(age) < 0.0001, ****p(genotype) = 0.0001, ***p(interaction) = 0.0003), with significantly increased expression in *Mir324* KO hippocampus at PD1 (Sidak’s multiple comparisons, *p = 0.0347) and PD 14 (****p < 0.0001). On average, Kv4.2 protein expression is increased at PD45-60 in *Mir324* KO, corroborating earlier findings that miR-324-5p negatively regulates Kv4.2 expression (Gross et al., 2016; Tiwari et al., 2019). Increased mRNA expression that is not associated with increased protein expression indicates control at the level of Kv4.2 mRNA translation.

### Mir324 KO alters hippocampal MAP2 protein and mRNA expression differentially across development

**Figure 6:**
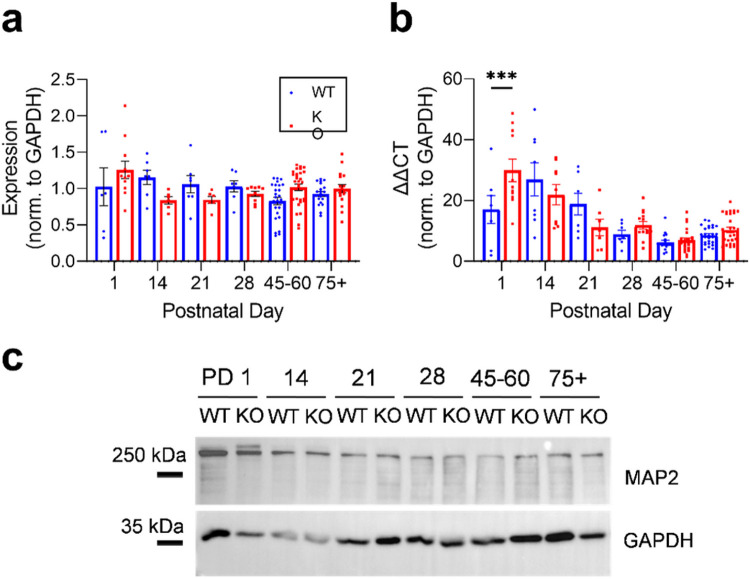
Hippocampal MAP2 protein and mRNA are differentially expressed across development and affected by *Mir324* KO. (A) Significant interaction of age and genotype on MAP2 protein expression (two-way ANOVA, **p(interaction)=0.009, p(age)=0.144, p(genotype)=0.64; Sidak’s multiple comparisons, all>0.05). (B) Interaction of age and genotype on MAP2 mRNA expression (two-way ANOVA, **p(interaction)<0.001, ****p(age)<0.0001, p(genotype)=0.4497; Sidak’s multiple comparisons, **p(PD 1)=0.001). (C) Western blot example images for WT and KO hippocampal tissue at each time point. Error bars are SEM. Full blots are shown in Supplemental Figure 1, n values and lack of sex differences are shown in Supplemental Tables 2 and 3.

To further elucidate potential mechanisms underlying the differential dendritic spine development in *Mir324* KO, we analyzed MAP2 protein and mRNA expression. MAP2 is a microtubule associated protein localized to neuronal dendrites that regulates dendritic spine structure and function (Y. Kim et al., 2020). Interestingly, though we found no effect of age or genotype on MAP2 protein expression in the hippocampus, we did find a significant interaction between age and genotype in protein expression (Fig. 6A; two-way ANOVA, p(age) = 0.144), p(genotype) = 0.64, **p(interaction) = 0.0088). Overall, MAP2 protein expression remained stable across development and did not vary significantly by genotype at any time point (Sidak’s multiple comparisons, all > 0.05). On average, MAP2 protein expression at PD45-60, was increased, in line with earlier findings (Parkins et al., 2023). Though protein expression remained stable across development, MAP2 mRNA expression varied by age (Fig. 6B; two-way ANOVA, ****p(age) < 0.0001, p(genotype) = 0.448, ***p(interaction) = 0.0009), with peak expression at PD1. Notably, this time point coincided with the time point when mRNA expression was significantly increased in *Mir324* KO hippocampi (Sidak’s multiple comparisons, **p = 0.001).

### Mir324 KO does not alter excitatory synapse composition on CA1 dendritic spines

**Figure 7:**
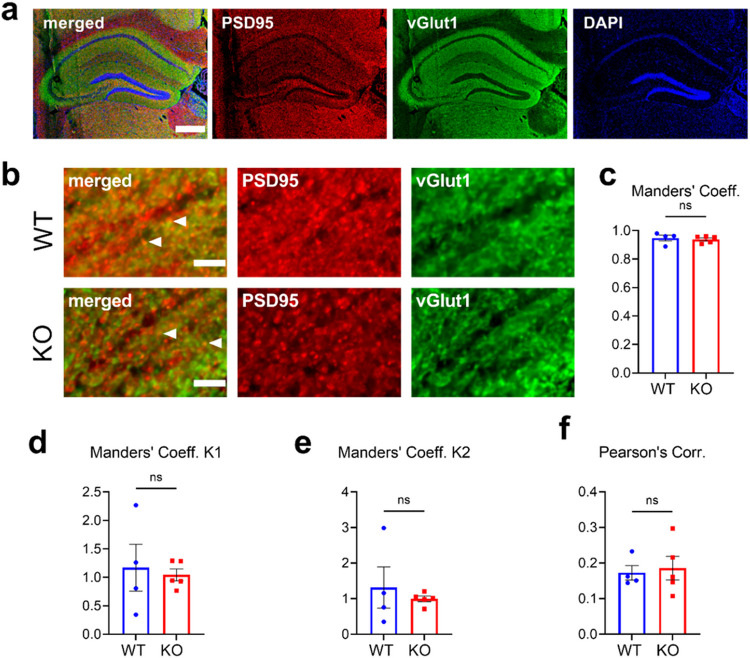
*Mir324* KO does not alter synapse formation on dendritic spines. (A) Representative image of hippocampal staining. White arrow indicates layer of hippocampal CA1 analyzed. Scale bar is 400 μm. (B) Example images of WT (top) and KO (bottom) CA1 apical dendrites. White arrows show examples of coexpression of both synaptic markers. Scale bar is 5 μm. (C) No difference was found in Manders’ coefficient, a measure of the percentage of overlap between the postsynaptic marker PSD95 and the presynaptic marker vGlut1 (unpaired t-test, p=0.6775). (D-E) No difference in the proportion of colocalized PSD95, reported as Manders’ coefficient K1 (E, unpaired t-test with Welch’s correction, p=0.784), or colocalized vGlut1, reported as Manders’ coefficient K2 (F, unpaired t-test with Welch’s correction, p=0.622), were observed by genotype. (F) Likewise, no significant effect of genotype on Pearson’s correlation was observed (unpaired t-test, p=0.762, n(WT)=4 mice, n(KO)=5 mice, an average of 7 images were analyzed per mouse). Error bars are SEM.

Dendritic spine density is not a measure of synaptic density, and the reduction in dendritic spine density observed in PD60 *Mir324* KO mice may be compensated for by increased synapse formation on dendritic spines. To assess excitatory synapses on dendrites, we performed immunostaining on PD60 hippocampi for the postsynaptic marker PSD95 and presynaptic marker vGlut1 (Fig. 7A,B). Using Manders’ coefficient analysis (Manders et al., 1993), we measured the proportion of colocalized signal between PSD95 and vGlut1 (Manders’ coefficient, Fig. 7C), the proportion of PSD95 that is colocalized with vGlut1 (Manders’ coefficient K1, Fig. 7D), and the proportion of vGlut1 colocalized with PSD95 (Manders’ coefficient K2, Fig. 7E) within the CA1 region. No differences in these measurements were found between *Mir324* KO and WT hippocampal neurons (Fig. 7C: unpaired t-test, p = 0.3445; Fig. 7D: unpaired t-test with Welch’s correction, p = 0.784; Fig. 7E: unpaired t-test with Welch’s correction, p = 0.622, n(WT) = 4 mice, n(KO) = 5 mice, an average of 7 images assessed per mouse). To account for any differences in background staining between images, we also measured the Pearson’s correlation coefficient, which is insensitive to background. No differences in Pearson’s correlation were found between genotypes (Fig. 7F: unpaired t-test, p = 0.762, n(WT) = 4 mice, n(KO) = 5 mice, an average of 7 images were analyzed per mouse). Overall, these results suggest that loss of miR-324-5p does not affect excitatory synapse composition on dendritic spines. Example images of immunostained hippocampi are shown in Fig. 7A-B.

## Discussion

Development of dendritic spines and synapses is a dynamic process, engaging a multitude of regulatory networks that vary over time (Calabrese et al., 2006; Grossman et al., 2010; Hu & Li, 2017). MicroRNAs are important synaptic regulators which are just beginning to be characterized. It is likely that they play a complex role in developmental synapse regulation, and that this role varies across development as the brain changes. Here, we show that a microRNA that has been recently implicated in dendritic spine density and morphology regulation (Parkins et al., 2023), miR-324-5p, is not only differentially expressed across development, but also may have distinct, developmental time point-specific regulatory roles. In particular, we show that deletion of the gene coding for miR-324-5p, *Mir324* alters the pattern of dendritic spine pruning over development, leading to a premature reduction in dendritic spine density that is compensated for later in development.

The molecular mechanisms underlying the effects of *Mir324* deletion on dendritic spines are unclear. The gene *Mir324* encodes both miR-324-5p and miR-324-3p; however, miR-324-5p is expressed primarily in the brain while miR-324-3p is expressed mainly in other tissues (Ludwig et al., 2016). The effects of *Mir324* deletion are, therefore, expected to be mainly mediated by loss of miR-324-5p, and could be caused by dysregulated expression of its targets or indirect effects on other key players in dendritic spine formation. We show that *Mir324* loss leads to changes in the expression of the dendritic protein MAP2, the synaptic protein PSD95, both of which are not experimentally confirmed targets of miR-324-5p, as well as the confirmed miR-324-5p target Kv4.2 across development. All three of these proteins are localized to dendrites and synapses and are involved in regulating dendritic morphology, synapse formation and synaptic strength (Berry & Nedivi, 2017; DeGiosio et al., 2022; J. Kim et al., 2007). Moreover, our unpublished RNA sequencing data (not shown) indicate that *Mir324* KO leads to aberrant expression of genes associated with cytoskeletal regulation, suggesting potential alterations in cytoskeletal stabilization. Indeed, we show that MAP2 protein expression is increased at select time points in the *Mir324* KO hippocampus. Future studies are needed to elucidate if changes in PSD95, MAP2 or Kv4.2 expression are causative of the premature dendritic spine loss in *Mir324* KO.

Our study is in line with several other studies that suggest that miR-324-5p regulates dendritic spine formation. MiRNA sequencing of the barrel cortex in a mouse model of associative memory revealed changes in the expression of numerous miRNAs and their targets, including miR-324-5p (Yan et al., 2016). Antagomir-induced silencing of miR-324-5p and miR-133a further demonstrated that concomitant loss of both miRNAs reduces dendritic spine formation in associative memory (Feng et al., 2017; Lei et al., 2017; Wu et al., 2020). Others suggested that miR-324-5p is involved in synapse pruning, with the loss of astrocytic miR-324-5p leading to reduced density in cultured neurons from Dicer KO mice (Sun et al., 2019). Of note, adult *Mir324* knockout mice have a lower proportion of thin, unstable spines supporting a role in regulating dendritic spine dynamics (Parkins et al., 2023). A limitation of our study is that we only assessed dendritic spine density at select time points across development, which provides a snapshot at a certain time but does not consider rapid changes caused by differential effects of, for example, environmental stressors on *Mir324* KO mice. Future studies are needed to assess the role of *Mir324-5p* in short- and long-term dendritic spine dynamics.

Changes in dendritic spine density with miR-324-5p loss across development could result from aberrant spine stabilization, formation, or pruning. Synaptic refinement and the highly regulated pruning of synapses is essential for proper neurodevelopment. By studying the effect of *Mir324* KO on dendritic spine density at different time points, we identified that miR-324-5p loss leads to earlier reduction in dendritic spine density. This suggests that miR-324-5p plays a role in stabilizing dendritic spines prior to final synaptic refinement. Previous work shows that miR-324-5p expression is essential for the maintenance of long-term potentiation (Parkins et al., 2023), a process that requires a morphological shift in dendritic spines dependent upon translocation of the protein MAP2 (Y. Kim et al., 2020). Dysregulated (over)expression of MAP2 has also been suggested to contribute to dendritic abnormalities, including short and sparse dendrites (Hurtado et al., 2015; Kaufmann et al., 2000). This effect is likely due to MAP2-mediated inhibition of polymerization and neurite formation (Kaufmann et al., 2000). Altogether, this suggests that miR-324-5p may regulate dendritic spine stability and pruning. Further research is necessary to shed light on the mechanism(s) by which miR-324-5p regulates dendritic spines.

Our results indicate changes in Kv4.2, MAP2 and PSD95 protein expression in the hippocampus across development and when *Mir324* is deleted, but it is not clear if proteins are differentially expressed in hippocampal subregions. Kv4.2 is enriched in the hippocampal CA1 subregion in adult mice (Rhodes et al., 2004), but it is unknown how subregion-specific expression levels change depending on age. MAP2 is expressed in the entire hippocampus proper but previous research suggests that subregional expression may change in older rats (Di Stefano et al., 2001). The hippocampal subregion-specific expression of the three proteins depending on age and *Mir324* genotype will have to be assessed in future studies.

We also show differences in age- and genotype-dependent mRNA and protein expression of Kv4.2, MAP2 and PSD95. This could be caused by changes in mRNA degradation, mRNA translation and protein stability across development which may be altered in *Mir324* KO. All three mRNAs have been shown to be present in dendrites and are bound to and/or translationally regulated by the Fragile X Messenger Riboprotein (FMRP) (Gross et al., 2011; Hale et al., 2021; Lee et al., 2011; Muddashetty et al., 2011). This suggests extensive post-transcriptional control beyond microRNA-induced silencing that could be altered across development and by genotype. Of note, we detected reduced FMRP expression in the hippocampus of adult *Mir324* KO mice (unpublished), which could underlie the differential regulation of mRNA and protein levels. Differential expression across subregions of the hippocampus may also account for some mismatch in the protein and mRNA expression results, with mRNA depletion in areas with high protein expression or imbalanced accumulation of mRNA or protein in specific subregions (Roy & Jacobson, 2013). Such changes would be masked in our subregion-unspecific approach. Kv4.2 turnover, for example, has been shown to vary depending on subcellular localization (Nestor & Hoffman, 2012), and protein longevity varies by cellular demands (West, 2019). Lastly, excitatory post-synapses vary greatly in the stability of PSD95, and synapses with diverse PSD95 protein stabilities are differentially distributed by subregion, age, and in disease (Bulovaite et al., 2022), which could contribute to our findings.

Other cell types in the brain, in addition to neurons, may also be involved in miR-324-5p-mediated dendritic spine regulation across development. MiR-324-5p is expressed in both neurons and astrocytes (Sun et al., 2019). Glial-neuron interactions play an integral role in determining spine density and synapse formation (Allen, 2014; Liddelow & Barres, 2017; Ota et al., 2013; Sun et al., 2019). Our prior studies suggest an important role of miR-324-5p in neurons by showing that miR-324-5p regulates neuronal excitability, seizure susceptibility and synaptic plasticity through the neuronal potassium channel Kv4.2 (Gross et al., 2016; Parkins et al., 2023; Tiwari et al., 2019). Sun et al. (2019) found that astrocytic miR-324-5p regulates synaptic density *in vitro*, but only with concomitant loss of miR-324-5p and Dicer, an essential protein for microRNA biogenesis (Sun et al., 2019). Together, these studies suggest that miR-324-5p affects neuronal morphology through both astrocytic and neuronal mechanisms.

MicroRNA are emerging as important synaptic regulators in processes like development, learning and memory, and in disease. MiR-324-5p loss leads to reduced dendritic spine density in the hippocampal CA1 (Parkins et al., 2023), but this effect varies significantly by age (Fig. 3). MicroRNA regulation at the synapse is complex. It likely involves multiple networks, biological pathways, and mechanisms, all of which can vary. Moreover, microRNA function is context-dependent, and may change with recent experiences. Other factors, such as disease, stress, subregion, and environmental enrichment, may all determine microRNA function at the synapse. Altogether, this study emphasizes the importance of greater context and control in the study of microRNA, especially in the highly dynamic environment of the synapse.
